# Variability of the response to immunotherapy among subgroups of patients with multiple sclerosis

**DOI:** 10.1111/ene.15706

**Published:** 2023-02-16

**Authors:** Ibrahima Diouf, Charles B. Malpas, Sifat Sharmin, Izanne Roos, Dana Horakova, Eva Kubala Havrdova, Francesco Patti, Vahid Shaygannejad, Serkan Ozakbas, Guillermo Izquierdo, Sara Eichau, Marco Onofrj, Alessandra Lugaresi, Raed Alroughani, Alexandre Prat, Marc Girard, Pierre Duquette, Murat Terzi, Cavit Boz, Francois Grand'Maison, Sherif Hamdy, Patrizia Sola, Diana Ferraro, Pierre Grammond, Recai Turkoglu, Katherine Buzzard, Olga Skibina, Bassem Yamout, Ayse Altintas, Oliver Gerlach, Vincent van Pesch, Yolanda Blanco, Davide Maimone, Jeannette Lechner‐Scott, Roberto Bergamaschi, Rana Karabudak, Gerardo Iuliano, Chris McGuigan, Elisabetta Cartechini, Michael Barnett, Stella Hughes, Maria José Sa, Claudio Solaro, Ludwig Kappos, Cristina Ramo‐Tello, Edgardo Cristiano, Suzanne Hodgkinson, Daniele Spitaleri, Aysun Soysal, Thor Petersen, Mark Slee, Ernest Butler, Franco Granella, Koen de Gans, Pamela McCombe, Radek Ampapa, Bart Van Wijmeersch, Anneke van der Walt, Helmut Butzkueven, Julie Prevost, L. G. F. Sinnige, Jose Luis Sanchez‐Menoyo, Steve Vucic, Guy Laureys, Liesbeth Van Hijfte, Dheeraj Khurana, Richard Macdonell, Riadh Gouider, Tamara Castillo‐Triviño, Orla Gray, Eduardo Aguera‐Morales, Abdullah Al‐Asmi, Cameron Shaw, Norma Deri, Talal Al‐Harbi, Yara Fragoso, Tunde Csepany, Angel Perez Sempere, Irene Trevino‐Frenk, Jan Schepel, Fraser Moore, Tomas Kalincik

**Affiliations:** ^1^ Department of Medicine CORe, University of Melbourne Melbourne Victoria Australia; ^2^ Department of Neurology Neuroimmunology Centre, Royal Melbourne Hospital Melbourne Victoria Australia; ^3^ Department of Neurology and Center of Clinical Neuroscience, First Faculty of Medicine Charles University in Prague and General University Hospital Prague Czech Republic; ^4^ Department of Medical and Surgical Sciences and Advanced Technologies GF Ingrassia Catania Italy; ^5^ Isfahan University of Medical Sciences Isfahan Iran; ^6^ Dokuz Eylul University Konak/Izmir Turkey; ^7^ Hospital Universitario Virgen Macarena Seville Spain; ^8^ Department of Neuroscience, Imaging, and Clinical Sciences D'Annunzio University Chieti Italy; ^9^ IRCCS Istituto delle Scienze Neurologiche di Bologna Bologna Italy; ^10^ Dipartimento di Scienze Biomediche e Neuromotorie Università di Bologna Bologna Italy; ^11^ Division of Neurology, Department of Medicine Amiri Hospital Sharq Kuwait; ^12^ CHUM Mississippi Center and University of Montreal Montreal Quebec Canada; ^13^ School of Medicine Ondokuz Mayis University Samsun Turkey; ^14^ KTU Medical Faculty, Farabi Hospital Trabzon Turkey; ^15^ Neuro Rive‐Sud Quebec City Quebec Canada; ^16^ Neurology Kasr Al Ainy MS Research Unit Cairo Egypt; ^17^ Department of Neuroscience Azienda Ospedaliera Universitaria Modena Italy; ^18^ CISSS Chaudière‐Appalache, Levis Sainte‐Marie Quebec Canada; ^19^ Haydarpasa Numune Training and Research Hospital Istanbul Turkey; ^20^ Central Clinical School Monash University Melbourne Victoria Australia; ^21^ Nehme and Therese Tohme Multiple Sclerosis Center American University of Beirut Medical Center Beirut Lebanon; ^22^ Department of Neurology, School of Medicine Koc University Istanbul Turkey; ^23^ Koc University Research Center for Translational Medicine Istanbul Turkey; ^24^ Zuyderland Medical Center Sittard‐Geleen the Netherlands; ^25^ Cliniques Universitaires Saint‐Luc, Louvain Brussels Belgium; ^26^ Center of Neuroimmunology, Service of Neurology, Hospital Clinic of Barcelona Barcelona Spain; ^27^ Garibaldi Hospital Catania Italy; ^28^ School of Medicine and Public Health University of Newcastle Newcastle New South Wales Australia; ^29^ IRCCS Mondino Foundation Pavia Italy; ^30^ Hacettepe University Ankara Turkey; ^31^ Ospedali Riuniti di Salerno Salerno Italy; ^32^ St Vincent's University Hospital Dublin Ireland; ^33^ UOC Neurologia, Azienda Sanitaria Unica Regionale Marche–AV3 Macerata Italy; ^34^ Brain and Mind Centre Sydney New South Wales Australia; ^35^ Royal Victoria Hospital Belfast UK; ^36^ Department of Neurology Centro Hospitalar Universitário de São João Porto Portugal; ^37^ Department of Neurology ASL3 Genovese Genoa Italy; ^38^ Department of Rehabilitation ML Novarese Hospital Moncrivello Genoa Italy; ^39^ Departments of Medicine and Clinical Research, Neurologic Clinic and Policlinic University Hospital and University of Basel Basel Switzerland; ^40^ Trias and Pujol Brothers University Hospital Badalona Spain; ^41^ Hospital Italiano Buenos Aires Argentina; ^42^ Liverpool Hospital Sydney New South Wales Australia; ^43^ Azienda Ospedaliera di Rilievo Nazionale San Giuseppe Moscati Avellino Avellino Italy; ^44^ Bakirkoy Education and Research Hospital for Psychiatric and Neurological Diseases Istanbul Turkey; ^45^ Aarhus University Hospital Aarhus Denmark; ^46^ Flinders University Adelaide South Australia Australia; ^47^ Monash Medical Centre Melbourne Victoria Australia; ^48^ Department of Medicine and Surgery University of Parma Parma Italy; ^49^ Groene Hart Ziekenhuis Gouda the Netherlands; ^50^ University of Queensland Brisbane Queensland Australia; ^51^ Nemocnice Jihlava Jihlava Czech Republic; ^52^ Rehabilitation and MS Center Overpelt and Hasselt University Hasselt Belgium; ^53^ Department of Neurology Alfred Hospital Melbourne Victoria Australia; ^54^ Austin Health Melbourne Victoria Australia; ^55^ CSSS Saint‐Jerome Saint‐Jerome Quebec Canada; ^56^ Medical Center Leeuwarden Leeuwarden the Netherlands; ^57^ Hospital de Galdakao‐Usansolo Galdakao Spain; ^58^ Westmead Hospital Sydney New South Wales Australia; ^59^ University Hospital Ghent Ghent Belgium; ^60^ Postgraduate Institute of Medical Education and Research Chandigarh India; ^61^ Department of Neurology Razi Hospital Manouba Tunisia; ^62^ Instituto de Investigacion Sanitaria Biodonostia, Hospital Universitario Donostia San Sebastian Spain; ^63^ South East Trust Belfast UK; ^64^ University Hospital Reina Sofia Cordoba Spain; ^65^ Department of Medicine Sultan Qaboos University Hospital Seeb Oman; ^66^ University Hospital Geelong Geelong Victoria Australia; ^67^ Hospital Fernandez Buenos Aires Argentina; ^68^ Neurology Department King Fahad Specialist Hospital–Dammam Dammam Saudi Arabia; ^69^ Universidade Metropolitana de Santos Santos Brazil; ^70^ Department of Neurology, Faculty of Medicine University of Debrecen Debrecen Hungary; ^71^ Hospital General Universitario de Alicante Alicante Spain; ^72^ Instituto Nacional de Ciencias Medicas y Nutricion Salvador Zubiran Mexico City Mexico; ^73^ Waikato Hospital Hamilton New Zealand; ^74^ Jewish General Hospital Montreal Quebec Canada

**Keywords:** EDSS, immunotherapy, marginal structural model, multiple sclerosis, relapse

## Abstract

**Background and purpose:**

This study assessed the effect of patient characteristics on the response to disease‐modifying therapy (DMT) in multiple sclerosis (MS).

**Methods:**

We extracted data from 61,810 patients from 135 centers across 35 countries from the MSBase registry. The selection criteria were: clinically isolated syndrome or definite MS, follow‐up ≥ 1 year, and Expanded Disability Status Scale (EDSS) score ≥ 3, with ≥1 score recorded per year. Marginal structural models with interaction terms were used to compare the hazards of 12‐month confirmed worsening and improvement of disability, and the incidence of relapses between treated and untreated patients stratified by their characteristics.

**Results:**

Among 24,344 patients with relapsing MS, those on DMTs experienced 48% reduction in relapse incidence (hazard ratio [HR] = 0.52, 95% confidence interval [CI] = 0.45–0.60), 46% lower risk of disability worsening (HR = 0.54, 95% CI = 0.41–0.71), and 32% greater chance of disability improvement (HR = 1.32, 95% CI = 1.09–1.59). The effect of DMTs on EDSS worsening and improvement and the risk of relapses was attenuated with more severe disability. The magnitude of the effect of DMT on suppressing relapses declined with higher prior relapse rate and prior cerebral magnetic resonance imaging activity. We did not find any evidence for the effect of age on the effectiveness of DMT. After inclusion of 1985 participants with progressive MS, the effect of DMT on disability mostly depended on MS phenotype, whereas its effect on relapses was driven mainly by prior relapse activity.

**Conclusions:**

DMT is generally most effective among patients with lower disability and in relapsing MS phenotypes. There is no evidence of attenuation of the effect of DMT with age.

## INTRODUCTION

Immunomodulation is the mainstay of treatment of multiple sclerosis (MS). Studies have suggested that immunotherapy reduces relapses and disability worsening and delays mortality [1, 2, 3, 4, 5, 6, 7]. However, the effectiveness of these disease‐modifying therapies (DMTs) may differ according to patient demographic and clinical characteristics, such as age or phase of the disease [8]. Whereas the presently approved DMTs have demonstrated effect on the course of relapsing–remitting MS, their effect in progressive MS has been considerably smaller [8, 9, 10, 11].

Understanding how demographic and clinical profiles modify the effectiveness of DMTs is important for the correct assessment of their risk–benefit ratios and appropriate use in specific clinical scenarios. Apart from the differences in efficacy of DMTs in relapsing versus progressive MS [8], understanding of demographic and clinical modifiers of the effectiveness of DMTs is limited. The aim of this study is to assess whether the effectiveness of DMT is influenced by previous disease activity, disability, age, MS duration, or disease phenotype.

Standard methods for controlling confounding factors are inappropriate when a time‐varying confounder is affected by previous treatment status, because over time such a confounder will also play the role of a mediator of treatment effect [12]. Robins et al. reported that standard modeling approaches may be biased whether or not one adjusts for confounder history in the analysis, (i) in the presence of a time‐dependent covariate that is a risk factor for, or predictor of, the event of interest and also predicts subsequent exposure; and (ii) past history predicts the subsequent level of the covariate [13]. For example, a history of relapse poses a risk for disease progression and future relapses and is also associated with subsequent treatment choices. We have therefore used marginal structural models (MSMs), recently adopted in MS research, to compare risks of relapses and disease progression between treated and untreated patients with MS [14, 15]. In particular, we have evaluated how effectiveness of DMTs varies according to patients' demographic and clinical profiles. The adopted methodology mitigates the effects of time‐varying confounders that are also influenced by prior evolution of the studied outcomes and previous treatment exposure [12].

## MATERIALS AND METHODS

### Study design

In this study, we have compared disability outcomes and relapse frequencies in treated versus untreated states.

Participants' first visit with disability information was used as a study baseline (Time 0). Patients were allowed to transition from “nontreated” to “treated” status. However, follow‐up was censored at first treatment discontinuation, meaning that patients could not revert to an “untreated” period after a “treated” period in the analysis, to minimize unmeasured bias driving the decision to stop therapy (Figure S3). In addition to this first approach (Approach A), we conducted two sensitivity analyses. In Approach B, patients' MS onset date was used as the study baseline, with no additional rebaselining. Patients were allowed to switch freely between treated and untreated status without censoring and with the time recorded relative to the date of MS onset (Figure S4). In Approach C, patients' first visit with disability information was used as the baseline with a baseline reset at each change in treatment state. Approach C also allowed switching between the treated and untreated states in both directions (Figure S5).

### Study population

Data used for this study were obtained from MSBase, an international MS registry approved by the Melbourne Health Human Research Ethics Committee (registered with WHO ACTRN12605000455662). Participants were required to provide informed consent as per the local regulations. We extracted data from 61,810 patients from 135 centers across 35 countries in March 2019 (recorded retrospectively since the 1970s and prospectively since 2003). MS centers recorded data prospectively as part of clinical practice following standard data quality procedures. We excluded centers in the lowest quintiles of data quality or generalizability scores [16]. Patients with clinically isolated syndrome or definite MS who satisfied the minimum data requirements were included in this study. The minimum required data consisted of: follow‐up ≥ 1 year, ≥3 Expanded Disability Status Scale (EDSS) scores with ≥1 score recorded per year, and a minimum dataset (sex, date of birth, MS onset date, MS course, EDSS score, magnetic resonance imaging [MRI] status in the past 12 months, pregnancy status). Only patients with relapsing–remitting MS or clinically isolated syndrome were included in the primary analysis (censoring follow‐up at the conversion to secondary progressive MS as diagnosed by treating neurologists). Secondary analysis included patients with relapsing and progressive MS phenotypes. For individual patients, only time from the first to the last recorded EDSS was included in this study.

### Variables of interest

Relapses were recorded by treating neurologists when patients presented new symptoms or exacerbation of existing symptoms persisting for ≥24 h, in the absence of concurrent illness/fever, and occurring ≥30 days after a previous relapse. Presence/absence of new or enlarging T2 hyperintense lesions or contrast‐enhancing lesions on cerebral MRI was reported by treating neurologists.

Follow‐up time was divided into intervals (bins) of 6 months. Potential confounders and intermediates of treatment effect were captured in each of these follow‐up epochs. Treatment status was updated at each bin, where patients were classified as “treated” (290,051 bins [78% of bins]) if DMTs were recorded for ≥15 days; otherwise they were classified as “untreated” (80,667 bins). This conservative classification enabled us to classify any treatment effect as a “treated” state, including extended effect of previously discontinued therapy and incipient effect of newly commenced therapy, while choosing to underestimate rather than overestimate the magnitude of the overall treatment effect [15, 17, 18]. Disability was quantified with the EDSS, excluding scores obtained <30 days after a relapse. For a minority of relapses, a longer time may be required before disability returns to the original or new baseline level. This few relapses do not typically have a substantial effect on the overall disability outcomes [19]. Neurostatus EDSS certification was required at the participating centers [20]. Where no new disability data were recorded during a 6‐month period, the last previously recorded disability score was carried over. Only 27% of EDSS values were carried over to periods without EDSS records. In our previous work in this cohort, 55% of 3‐month bins contained recorded EDSS information, with high correlation among the consecutive bins in which EDSS was captured (*r* = 0.95). The number of relapses within each bin were counted, and an annualized relapse rate was calculated.

Disability worsening was defined as an increase in EDSS by 1 step (1.5 step if baseline EDSS = 0 and 0.5 steps if baseline EDSS > 5.5) confirmed by subsequent EDSS scores over ≥12 months, as >80% of such events correspond to long‐term worsening of disability [15]. Disability improvement was defined as a decrease in EDSS by 1 step (1.5 steps if baseline EDSS ≤ 1.5 and 0.5 steps if baseline EDSS > 6) confirmed over ≥12 months. No carryover EDSS scores were utilized in calculating confirmed disability endpoints. Presence or absence of relapses during each 6‐month interval was defined as a binary variable.

### Statistical methods

In this study, we have compared treated versus untreated patients (pseudocohorts) within patient groups defined by their demographic and clinical characteristics [15]. We used MSMs to estimate the “per‐protocol” causal effects of DMTs on the studied outcomes. Inverse probability of treatment weights was calculated for each follow‐up bin of 6 months to reflect the extent to which a certain observation was under‐ or overrepresented with respect to a pseudopopulation in which these characteristics are balanced across treatment groups [12]. In practice, treatment weights are calculated based on the inverse of each participant's probability of receiving treatment at each 6‐month interval given their covariates history (age, sex, pregnancy status, treatment history, history of relapses, MS duration, EDSS, date of birth, and MS course). Figure S1 depicts hypothetical relationships between variables included in the calculation of the weights.

$$w_{\mathrm{ij}}=\prod_{k=0}^{j}\frac{P\left(A_{\mathrm{ik}}=a_{\mathrm{ik}}\;,|,A_{i\left(k-1\right)},=,a_{i\left(k-1\right),},\;,S_{i},=,s_{i},\;\right)}{P\left(A_{\mathrm{ik}}=a_{\mathrm{ik}}\;,|,\;,A_{i\left(k-1\right)},=,a_{i\left(k-1\right)},,,T_{\mathrm{ik}},=,t_{\mathrm{ik}},,,S_{i},=,s_{i},\;\right).}$$

where $w_{\mathrm{ij}}$ represents the stabilized weight for patient *i* at time *j. A* is the treatment status at time *k*, and *S* represents both fixed covariates and the baseline stabilizing variables (sex, MS duration at first visit, date of birth). *T* represents time‐dependent covariates (age, pregnancy status, treatment history, history of relapses, MS duration, EDSS, MS course) [14]. Date of birth and MS onset date are included in the model of weights in addition to age and MS duration to capture long‐term changes in the availability of DMTs and MS management. The weights reflect the probability of patients' treatment status at each 6‐month period given their demographic and disease history. Interactions of demographic and clinical variables with DMT were studied as the primary terms of interest to quantify the effect of patients' characteristics on the effectiveness of MS therapy. The respective demographic or clinical variable was not included in the model of weights where its interaction with DMT status was investigated in the outcomes model. The distribution of the weights was assessed for normality and absence of extreme values, and to rule out model misspecification (Figure S2). After calculating the weights, we used Cox proportional hazards models with the inverse probability of treatment weights (MSM Cox model) to compare the cumulative hazards of disability improvement, disability worsening, and relapses between the treated and untreated pseudocohorts. The analyses were performed using R version 3.6.1.

## RESULTS

Among 61,810 patients of the MSBase registry, 26,329 participants met the eligibility criteria for the full cohort (including all MS phenotypes), consisting of the relapsing cohort (24,344 patients; Table 1) and progressive cohort (1985 patients). Patients' demographic features at first visit (mean age = 36 years [SD = 11 years], 71% female) were in keeping with the known epidemiology of MS (Table 1) [21]. The median duration of follow‐up among the included participants was 8.8 years. Interferon beta was the most common DMT at the first EDSS visit (35% of all participants). Although the use of high‐efficacy therapies was low at the first visit (10%), 22% of patients switched to these therapies during the follow‐up. When compared to participants included in this study, those who were excluded tended to be older, with longer MS duration at baseline and to present with more severe disability and fewer MRI lesions, and were less often treated with DMTs (Tables S1 and S2).

**TABLE 1:** Characteristics of the study population at the first visit

Characteristic	Relapsing MS, *n* = 24,344	Full cohort, *N* = 26,329
Age at first visit, years, mean (SD)	35.5 (10.5)	36.3 (10.9)
Age at MS onset, years, mean (SD)	30.6 (9.70)	30.8 (9.84)
Female	17,408 (71.5%)	18,620 (70.7%)
Ms duration, years, median [Q1, Q3]	2.77 [0.75, 7.75]	3.08 [0.835, 8.54]
Disability		
EDSS 0–3.5	20,996 (86.2%)	21,449 (81.5%)
EDSS 4–5.5	2450 (10.1%)	3081 (11.7%)
EDSS 6–9.5	898 (3.7%)	1799 (6.8%)
MRI lesions in the past 12 months	1172 (4.8%)	1227 (4.7%)
MS course		
RR	20,599 (84.6%)	20,599 (78.2%)
CIS	3745 (15.4%)	3745 (14.2%)
PR	–	243 (0.9%)
SP	–	1196 (4.5%)
PP	–	546 (2.1%)
DMTs at first visit		
None	10,171 (41.8%)	11,159 (42.4%)
Interferon beta	8745 (35.9%)	9288 (35.3%)
Glatiramer acetate	2280 (9.4%)	2419 (9.2%)
Natalizumab	1169 (4.8%)	1247 (4.7%)
Fingolimod	1036 (4.3%)	1102 (4.2%)
Dimethyl fumarate	353 (1.5%)	366 (1.4%)
Teriflunomide	310 (1.3%)	328 (1.2%)
Mitoxantrone	135 (0.6%)	251 (1.0%)
RCT	42 (0.2%)	52 (0.2%)
Rituximab	44 (0.2%)	48 (0.2%)
Cladribine	17 (0.1%)	20 (0.1%)
Daclizumab	16 (0.1%)	19 (0.1%)
Alemtuzumab	16 (0.1%)	16 (0.1%)
Ocrelizumab	10 (0.0%)	13 (0.0%)
AHSCT	–	1 (0.0%)
Switch to high‐efficacy DMT	5353 (22.0%)	5889 (22.4%)

Abbreviations: AHSCT, autologous haematopoietic stem cell transplantation; CIS, clinically isolated syndrome; PP, primary progressive; PR, progressive relapsing; RCT, randomised controlled trial; RR, relapsing–remitting; SP, secondary progressive; DMT, disease‐modifying therapy; EDSS, Expanded Disability Status Scale; MRI, magnetic resonance imaging; MS, multiple sclerosis.

Overall, DMTs were associated with 48% lower risk of relapses (hazard ratio [HR] = 0.52, 95% confidence interval [CI] = 0.45–0.60), 46% lower risk of disability worsening (HR = 0.54, 95% CI = 0.41–0.71), and 32% higher chance of disability improvement (HR = 1.32, 95% CI = 1.09–1.59; Figure S3). We conducted sensitivity analyses with different definitions of baseline and follow‐up (Figures S4 and S5). If patients were included from MS onset date without any restriction on follow‐up (Approach B), DMTs were associated with 47% lower risk of relapses, 36% lower risk of disability worsening, and 33% higher chance of disability improvement. If patients were included from the first EDSS visit with patients' rebaselining at each change in treatment status (Approach C), DMTs were associated with 32% lower risk of relapses 24% lower risk of disability worsening, and 38% higher chance of disability improvement.

**FIGURE 1:**
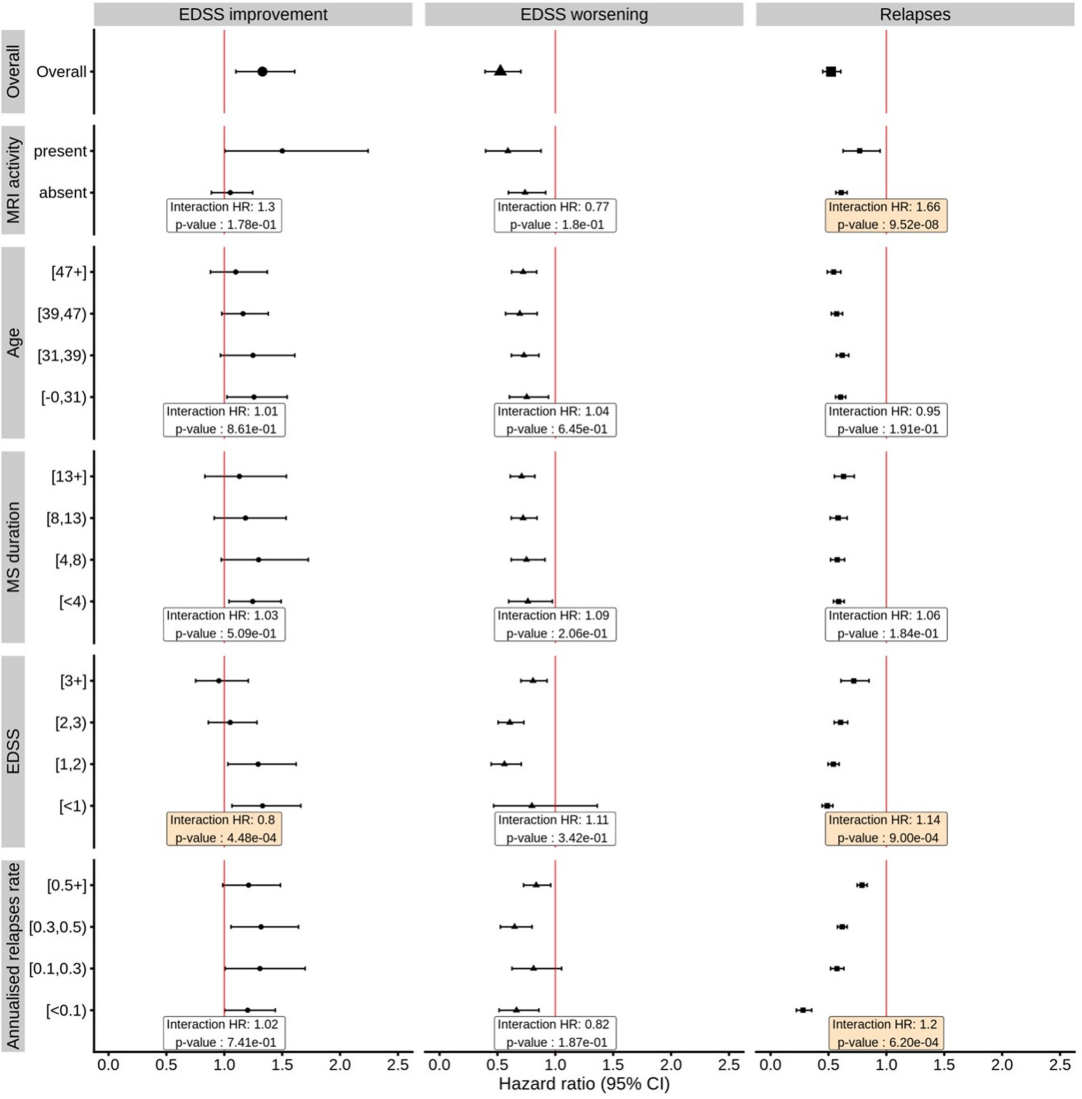
Weighted modifiers of the effects of disease‐modifying therapy (DMT) on disability improvement, disability worsening, and relapses. Results were estimated from stratified marginal structural Cox models using data from the relapsing multiple sclerosis (MS) cohort (clinically isolated syndrome and relapsing–remitting). Weights for the marginal structural model were calculated from a logistic model with treatment status as a dependent variable; independent variables included fixed covariates and the baseline stabilizing variables (sex, MS duration at first visit, date of birth) and time‐dependent covariates (age, pregnancy status, treatment history, history of relapses, MS duration, Expanded Disability Status Scale [EDSS], MS course). The “Overall” row in the figure displays the overall effects of treatment. Points and error bars represent hazard ratios (HRs) for associations of DMT with the studied outcomes in different patient groups. The boxes show HRs and *p*‐values for the interactions between DMT and demographic and clinical characteristics indicated along the y‐axis. CI, confidence interval; MRI, magnetic resonance imaging

The main aim of this study was to explore the effect of patient age, MS duration, EDSS, annualized relapse rate, and MRI activity (present/absent) on the effectiveness of DMTs (Figure 1).

We did not find evidence for the effect of age on the effectiveness of DMTs on MS relapses (HR = 0.95, 95% CI = 0.89–1.02). The magnitude of the difference in the effectiveness of DMTs was negligible among the compared age groups (range in mean DMT effectiveness = 40%–46%; Figure 1). This was further supported by the observation that MS duration was not associated with change in the effectiveness of DMTs on relapses (Figure 1). We did not find evidence of interactions between DMTs and age or MS duration for disability worsening and disability improvement (Figure 1). However, the results from the stratified MSM suggested that disability improvement may be more likely among younger patients (26% in those aged <31 years, 25% in those aged 31–38 years, 16% in those aged 39–46 years, and 10% in those aged ≥47 years). Similarly, disability improvement tended to be more common in those with a shorter MS duration at first visit (25% chance of improvement for MS duration < 4 years, 30% for MS duration = 4–8 years, 18% for MS duration = 8–14 years, and 13% for MS duration ≥14 years; Figure 1).

Interactions between DMT and EDSS were evident for relapses (HR = 1.14, 95% CI = 1.05–1.23), and disability improvement (HR = 0.80, 95% CI = 0.71–0.91, Figure 1). The results suggest a 14% increase in the difference in the hazard of relapses between treated and nontreated patients with each step increase in EDSS. They also show 20% decrease in the difference in the probability of disability improvement between treated and nontreated patients with each step increase in EDSS. When MSMs were stratified by quartiles of EDSS (Figure 1), DMTs appeared more effective at increasing disability improvement and at reducing relapses among patients with low EDSS compared to higher EDSS.

An interaction for the effectiveness of DMTs on relapses was observed with previous annualized relapse rate (HR = 1.20, 95% CI = 1.08–1.33). Stratified MSMs showed a more prominent effect of DMTs on reducing relapses in patients with lower prior annualized relapse rates (range of the apparent DMT effectiveness = 72%–21%).

Presence/absence of MRI activity modified the effect of DMTs on relapses (HR for interaction = 1.66, 95% CI = 1.38–2.00) but not on disability worsening and disability improvement. In analyses stratified by MRI activity, DMTs were associated with 41% lower risk of relapses in those without MRI activity (HR = 0.59, 95% CI = 0.40–0.88) versus 26% in those with MRI activity (HR = 0.74, 95% CI = 0.60–0.92).

**FIGURE 2:**
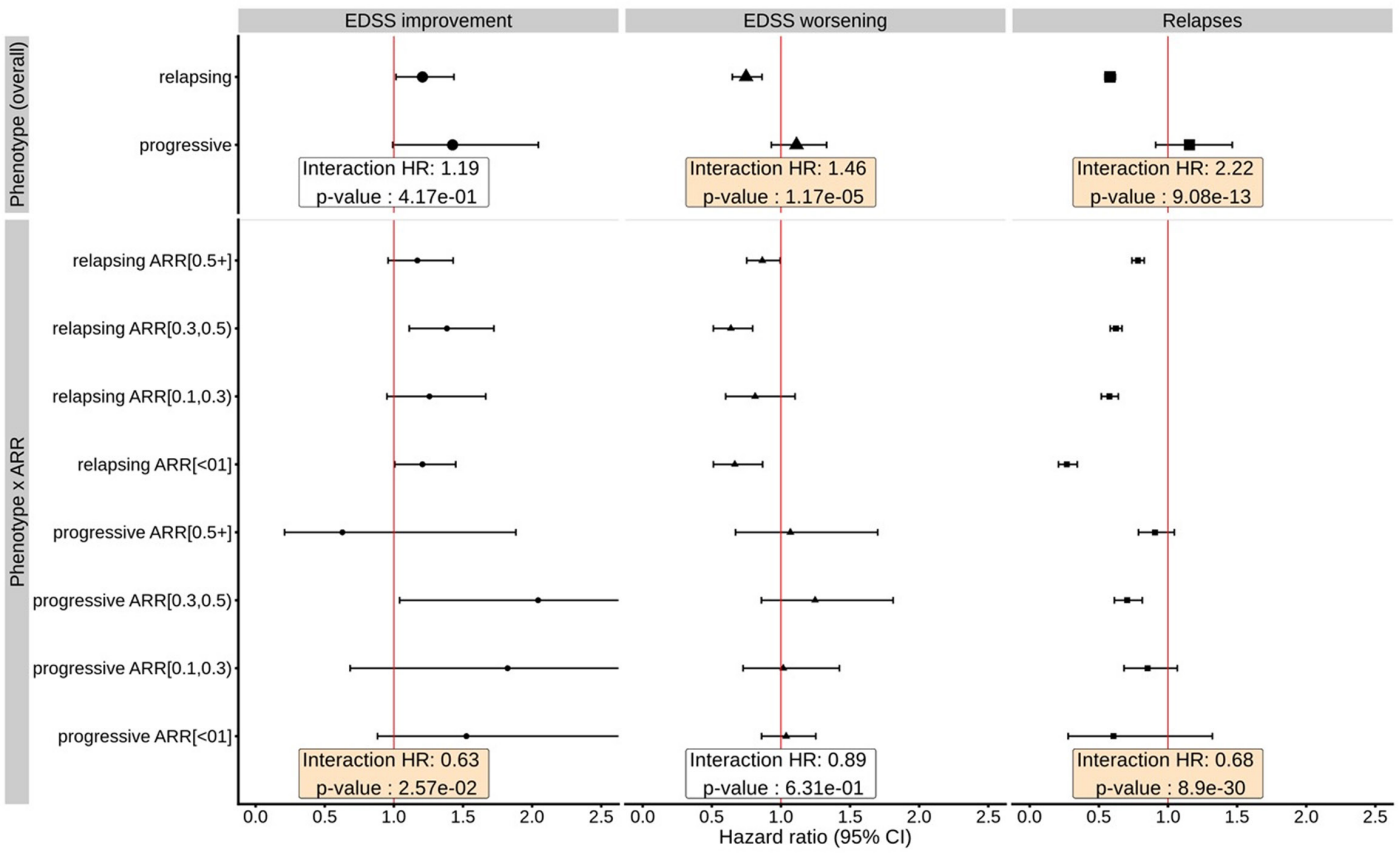
Weighted effects of disease‐modifying therapy (DMT) in relapsing and progressive multiple sclerosis (MS), showing data from the full cohort (including relapsing and progressive MS). Data are from marginal structural models. Weights for the marginal structural model were calculated from a logistic model with treatment status as a dependent variable; independent variables included fixed covariates and the baseline stabilizing variables (sex, MS duration at first visit, date of birth) and time‐dependent covariates (age, pregnancy status, treatment history, history of relapses, MS duration, Expanded Disability Status Scale [EDSS], MS course). The “Overall” rows in the figure display the overall effects of treatment. Hazard ratios (HRs) and *p*‐values for phenotype (overall) represent the interactions between DMT and disease phonotype. HRs and *p*‐values for phenotype × ARR represent a three‐way interaction between DMT, phenotype, and annualized relapse rate. The boxes show HRs and *p*‐values for the interactions between DMT and MS phenotype, and a three‐way interaction among DMT–MS phenotype–ARR. CI, confidence interval

In a pooled cohort of patients with relapsing and progressive MS phenotypes, disease phenotype was an important modifier of DMT effectiveness. Patients with relapsing MS derived more benefit than those with progressive MS in the effect of DMTs on disability worsening (HR for interaction = 1.46, 95% CI = 1.23–1.73; Figure 2). DMTs were associated with 25% lower risk of disability worsening (HR = 0.75, 95% CI = 0.65–0.86) in relapsing MS but not in progressive MS (HR = 1.11, 95% CI = 0.93–1.33). The effect of DMTs on EDSS worsening tended to depend more on MS phenotype rather than on relapse activity. In contrast, the effect of DMTs on relapses was more closely associated with the baseline relapse activity than disease phenotype (Figure 2). In both relapsing and progressive MS, we have seen a more pronounced reduction of relapse hazard among patients with lower baseline relapse activity (interaction term for DMT and MS phenotype: HR = 0.68, 95% CI = 0.58–0.79), even though the effect of DMTs reached formal statistical significance only in relapsing MS.

## DISCUSSION

DMTs lower the risk of relapses and disability worsening and, in some instances, improve the chance of disability improvement [15, 22]. In this study, we have established that the effectiveness of DMTs depends on patients' clinical and demographic characteristics. The effect of DMTs on preventing relapses and disability worsening is most pronounced among patients with lower disability. Similarly, DMTs are associated with an increased chance of disability improvement mainly among patients with mild disability (EDSS < 2). DMTs effectively prevent relapses in relapsing–remitting MS associated with both high and low degree of episodic inflammatory activity (presenting as relapses or active cerebral MRI). The effectiveness of DMTs on reducing disability worsening is affected more by MS phenotype, whereas the effectiveness of DMTs on suppressing relapses is more dependent on previous relapse frequency.

In our previous study, we demonstrated the use of MSMs in large observational datasets as a framework for evaluating overall treatment effectiveness across all disease stages and in broad, inclusive populations [15]. We have further expanded this approach by emulating a target trial (as shown in Table S3), in which the time patients spend treated with DMTs is compared to untreated time [23]. This is achieved by weighting of the included populations by their time‐dependent determinants of treatment status, which leads to comparisons of outcomes between two treatment states modeled in a single "pseudocohort" [24]. In the primary analysis, we have eliminated the potential bias due to informed treatment discontinuation by censoring follow‐up at the time when a treatment is stopped. However, patients are allowed to transition from the untreated to treated state. This approach might favor the effect of treatment and might, additionally, obscure delayed and cumulative effect of previous exposure to DMT. Therefore, we explored two additional designs. First, we have designed a pseudotrial in which patients' follow‐up was recorded relative to MS onset and where patients were allowed to transition between treatment states without restriction and without rebaselining the follow‐up after they switched between treatment states. The second, alternative design uses first disability time point as baseline, and patients' follow‐up time is rebaselined at each transition between the treated and untreated states. Both sensitivity analyses show similar magnitude of treatment effect as the primary analysis.

The effectiveness of DMTs is known to be lower in progressive MS forms than in relapsing MS [25]. We confirmed this phenomenon when we did not observe an overall effect of DMTs among patients with progressive MS in a pooled cohort. Moreover, secondary progressive MS is associated with more advanced disability [26, 27, 28]. Patients with low EDSS (<2, as well as younger age and shorter disease duration) were more likely to benefit from DMTs in terms of disability improvement, an observation that complements our earlier finding of recovery from disability due to MS therapy that occurs predominantly early after disease onset [15]. Other studies showed that disability is less likely to be reduced with DMTs in patients with EDSS > 4 [29, 30]. Importantly, even though the effectiveness of DMTs on disability worsening was reduced with increased EDSS, some effect on confirmed disability worsening was still seen among patients with more advanced disability. Our study therefore corroborates previous studies, which support the use of DMTs for recovery from disability in earlier, less advanced MS but suggest that DMTs continue to prevent disability accrual also in more advanced MS [10, 11, 31, 32, 33]. The EDSS has been previously reported to be less reflective of clinical deterioration in the high EDSS categories compared to low EDSS categories [34]. This may partly explain the attenuation of the effect of DMTs with increased EDSS.

Prior relapse activity was a more important determinant of the effect of DMTs on relapses than disease phenotype. Interestingly, we have observed a superior suppression of relapses by DMTs among patients with relapsing MS with lower relapse activity and with absence of recent radiological activity. This is most likely attributable to the high representation of low‐efficacy therapies (such as interferon β and glatiramer acetate), which may be sufficiently potent to suppress relapses in disease with a low level of episodic inflammation but may fail to suppress relapses in highly active disease [35, 36]. Furthermore, in many instances prior relapses occurred during DMT treatment, which is a recognized poor prognostic sign [37]. Finally, a group with consistently low frequency of relapses, which continues to receive their established DMTs, represents the cohort with well‐controlled disease. These patients will likely experience continued benefit from their present DMT.

This study did not find evidence for the effect of age on the effectiveness of DMTs. Patients aged 47 years or older continued to derive benefit from DMTs. This contrasts with a meta‐analysis of randomized clinical trials [38], which suggested that patients may cease to benefit from DMTs after 53 years of age. However, the meta‐analysis only used information about mean patient age within each trial and included a limited number of trials where mean patient age was 45 years (seven trials, only one trial with mean age > 50 years). Thus, the conclusion of the meta‐analysis was based on an extrapolation of treatment efficacy beyond the available data. Moreover, the trials with higher age averages are enriched by studies of DMTs in progressive MS, in which the effectiveness of DMTs is known to be low.

Furthermore, the results of this study suggest that timely initiation of DMTs, before patients accumulate severe and irreversible neurological disability, maximizes the benefit that patients will derive from exposure to these treatments [39]. This conclusion is supported by the results of several previous studies [40, 41, 42]. It also provides evidence in support of continued treatment across all sufficiently represented age groups among patients with relapsing disease.

This study is limited by the observational nature of the analyzed data. We have utilized a standardized data quality procedure to maximize the syntactic validity of the analyzed information [40]. By using MSMs, we were able to emulate target randomized trials of treated versus untreated pseudocohorts, which allow causal interpretations of the results [24]. MSMs allowed us to repeatedly rebalance the compared treated and untreated pseudocohorts for time‐varying confounders of the analyzed associations of DMTs with outcomes. Despite the substantial number of covariates that we have accounted for, unmeasured confounding may still be present (most notably MRI measures). It is therefore reassuring that the results of the overall treatment effectiveness were consistent across three different definitions of target trial. Multiple DMTs with different levels of efficacy were combined in the “treated” group of this study. We were, therefore, unable to compare effectiveness among multiple treatments simultaneously. Continuous variables were stratified in a way to allow sufficient representation of patients in each stratum rather than on clinical utility of the strata. Future research using MSMs will require application of methodology that allows simultaneous comparisons of treatments. Disability improvement due to DMT was mainly observed in the early years following the first presentation of MS. This result may suggest an exhaustion of compensatory mechanisms with increasing cumulative inflammatory damage and older age [15].

## CONCLUSIONS

Immunotherapy for MS is associated with reduction in relapse frequency and disability worsening, and increased chance of recovery of neurological function. DMTs are most effective in controlling relapsing MS before patients have accumulated substantial neurological disability. They continue to reduce disease activity and disability worsening even in patients with EDSS ≥ 3 and age 45 years or older. DMTs should therefore be initiated early, but should be considered across all age groups, with careful evaluation of individual benefit–risk ratios.

## AUTHOR CONTRIBUTIONS

Ibrahima Diouf designed the analysis, analyzed the data, and drafted the manuscript. Tomas Kalincik designed the concept, supervised the statistical analysis, and drafted the manuscript. All the other authors provided data and revised the manuscript.

## FUNDING INFORMATION

This study was financially supported by the National Health and Medical Research Council of Australia (1129189, 1140766, 1080518).

## CONFLICT OF INTEREST

The authors report the following relationships: speaker honoraria, advisory board or steering committee fees, research support, and/or conference travel support from Acthelion (E.K.H., R.Am.), Almirall (M.T., F.G., R.B., C.R.‐T., J.L.S.‐M.), Bayer (M.T., A.L., P.S., R.Am., C.B., J.L.‐S., V.v.P., R.B., D.S., R.Al., J.L.S.‐M., S.Ho., C.R.‐T., T.C‐T., M.S.), BioCSL (T.K.), Biogen (T.K., D.H., E.K.H., M.T., G.Iu., A.L., M.G., P.D., P.G., A.v.d.W., F.G'M., P.S., D.F., R.Am., C.B., J.L.‐S., V.v.P., F.G., R.B., R.Al., C.R.‐T., J.P., M.B., J.L.S.‐M., S.Ho., C.R.‐T., C.Sh., O.Gr., T.C‐T., M.S., H.B.), Biologix (R.Al.), Celgene (E.K.H.), Genpharm (R.Al.), Genzyme‐Sanofi (T.K., E.K.H., M.T., G.Iz., A.L., M.G., P.D., P.G., A.v.d.W., F.G'M., P.S., D.F., R.Al., M.T., C.B., J.L.‐S., V.v.P., F.G., R.B., D.S., C.R.‐T., J.P., M.B., J.L.S.‐M., S.Ho., O.Gr., H.B.), GSK (R.Al.), Merck/EMD (T.K., D.H., E.K.H., M.T., G.Iu., A.L., M.G., P.D., P.G., A.v.d.W., P.S., D.F., R.Am., M.T., C.B., J.L.‐S., V.v.P., F.G., R.B., D.S., R.Al., M.B., J.L.S.‐M., C.R.‐T., F.M., O.Gr., T.C‐T., M.S., H.B.), Mitsubishi (F.G'M.), Novartis (T.K., D.H., E.K.H., M.T., G.Iu., A.L., M.G., P.D., P.G., A.v.d.W., F.G'M., P.S., D.F., R.Am., M.T., C.B., J.L.‐S., V.v.P., F.G., R.B., D.S., R.Al., C.R.‐T., J.P., M.B., J.L.S.‐M., S.Ho., C.R.‐T., F.M., C.Sh., O.Gr., T.C‐T., M.S., H.B.), ONO Pharmaceuticals (F.G'M.), Roche (T.K., E.K.H., A.L., M.T., C.B., V.v.P.), Teva (T.K., D.H., E.K.H., M.T., G.Iz., A.L., M.G., P.D., P.G., F.G., P.S., D.F., M.T., C.B., J.L.‐S., V.v.P., R.B., D.S., R.Al., J.P., J.L.S.‐M., C.R.‐T., T.C‐T., M.S.), WebMD (T.K.).

## ETHICAL APPROVAL

The MS registry was approved by the Melbourne Health Human Research Ethics Committee (registered with WHO ACTRN12605000455662). Participants provided consent as per local regulations.

## CONSENT FOR PUBLICATION

This study complied with regulations and consent requirements for publication.

## CODE AVAILABILITY

Code written for analyzing data used for the article is available upon request from the corresponding author.

## Supporting information


DATA S1

## Data Availability

MSBase is a data processor, and warehouses data from individual principal investigators who agree to share their datasets on a project‐by‐project basis. Data access to external parties can be granted at the sole discretion of each MSBase Principal Investigator (the data controllers), who will need to be approached individually for permission.
