# Supplementary material for: Variability of the response to immunotherapy among subgroups of patients with multiple sclerosis
Source: Eur J Neurol. 2023 Feb 16;30(4):1014–24. doi: 10.1111/ene.15706 (PMC10946605; doi:10.1111/ene.15706)
Supplement: Supplementary file 1 — DATA S1 [file ENE-30-1014-s001.docx]

**Supplements**

**Supplementary table 1**. Included versus excluded patients when patients with relapsing MS phenotype are selected.

|  | | Excluded (n=38123) | | Included (n=24344) | |
| --- | --- | --- | --- | --- | --- |
|  | |  | |  | |
| Age first visit (years), Mean (SD) | | 39.1 (12.6) | | 35.5 (10.5) | |
| Female sex | | 26619 (69.8%) | | 17408 (71.5%) | |
| MS duration at baseline, Median [Q1, Q3] | | 4.19 [0.997, 11.0] | | 2.77 [0.75, 7.75] | |
| Disability | |  | |  | |
| EDSS 0-3.5 | | 21887 (57.4%) | | 20996 (86.2%) | |
| EDSS 4-5.5 | | 4940 (13.0%) | | 2450 (10.1%) | |
| EDSS 6-9.5 | | 5989 (15.7%) | | 898 (3.7%) | |
| Missing | | 5307 (13.9%) | | 0 (0%) | |
| Presence of MRI lesions in the last 12 months | | 898 (2.4%) | | 1172 (4.8%) | |
| Receiving disease modifying therapy at baseline | | 9567 (25.1%) | | 14173 (58.2%) | |

**Supplementary table 2**. Included versus excluded patients when all MS phenotypes are considered

|  | Excluded  (n=35481) | Included  (n=26329) |
| --- | --- | --- |
|  |  |  |
| Age first visit (years), Mean (SD) | 38.9 (12.6) | 35.8 (10.8) |
| Females | 24964 (70.4%) | 18620 (70.7%) |
| MS duration at baseline (years), Median [Q1, Q3] | 4.00 [0.925, 10.8] | 2.58 [0.668, 8.00] |
| Disability |  |  |
| EDSS 0-3.5 | 20881 (58.9%) | 21449 (81.5%) |
| EDSS 4-5.5 | 4235 (11.9%) | 3081 (11.7%) |
| EDSS 6-9.5 | 5058 (14.3%) | 1799 (6.8%) |
| Missing | 5307 (15.0%) | 0 (0%) |
| Presence of MRI lesions in the last 12 months | 820 (2.3%) | 1039 (3.9%) |
| Receiving disease modifying therapy | 8296 (23.4%) | 13843 (52.6%) |

**Supplementary table 3**: Summary of the study protocol

| **Protocol component** | **Description** |
| --- | --- |
| inclusion criteria | clinically isolated syndrome or definite multiple sclerosis |
| treatment strategies | patients may contribute 6-month periods to either the treated (where exposed to disease modifying therapy for ≥15 days during the given period) or the untreated pseudo-cohort (where exposure to disease modifying therapy during the given period <15 days) |
| assignment procedures | non-random assignation of therapy by treating neurologists |
| follow-up period | follow-up ≥1 year, ≥3 disability scores with ≥1 score recorded per year |
| outcomes | - 12-month confirmed disability worsening events (increase in EDSS by 1 step; 1.5 step if baseline EDSS=0 and 0.5 steps if baseline EDSS>5.5) - 12-month confirmed disability improvement events (decrease in EDSS by 1 step; 1.5 steps if baseline EDSS≤1.5 and 0.5 steps if baseline EDSS>6) - relapses |
| causal contrast of interest | per-protocol effect |
| analysis | proportional hazards models of multiple events with robust estimation of variance and inverse probability of treatment weights to adjust for fixed and time-dependent confounders and intermediates of outcomes; this analysis plan implies that data on the adjustment factors are available |

**Supplementary table 4:** List of contributors

The following contributors participated in data acquisition:

From AHEPA University Hospital, Thessaloniki, Greece

From Groene Hart Ziekenhuis, Gouda, Netherlands, Dr Freek Verheul

From Faculty of medicine Ain Shams University, Cairo, Egypt, Dr Magd Zakaria

From Centro Internacional de Restauracion Neurologica, Havana, Cuba, Dr Jose Antonio Cabrera-Gomez.

From MS Clinic, Hopital Tenon, Paris, France, Dr Etienne Roullet.

From University Hospital Nijmegen, Nijmegen, Netherlands, Dr Cees Zwanikken.

From Francicus Ziekenhuis, Roosendaal, Netherlands, Dr Leontien Den braber-Moerland.

From Zuyderland Medical Centre, Sittard-Geleen, Netherlands, Dr Oliver Gerlach.

From Rashid Hospital, Dubai, United Arab Emirates, Dr Jihad Inshasi.

From Clinica Modelo de Lanus, Lanus, Argentina, Dr Alfredo Firstenfeld.

From Instituto Rodriguez Alfici, Mendoza, Argentina, Dr Alberto Rodriguez Alfici.

From INEBA - Institute of Neuroscience Buenos Aires, Buenos Aires, Argentina, Dr Maria Laura Saladino.

From Instituto de Neurociencias Cordoba, Cordoba, Argentina, Dr Elizabeth Alejandra Bacile.

From HIGA Gral. San Martin LaPlata, La Plata, Argentina, Dr Vetere Santiago.

From Sanatorio Allende, Cordoba, Argentina, Dr Carlos Vrech.

From University of Western Australia, Nedlands, Australia, Dr Allan Kermode, Dr Marzena Fabis-Pedrini.

From Royal North Shore Hospital, Sydney, Australia, Dr John Parratt.

From St Vincents Hospital, Fitzroy, Melbourne, Australia, Dr Neil Shuey.

From Townsville Hospital, Townsville, Australia, Dr Mike Boggild.

From Royal Hobart Hospital, Hobart, Australia, Dr Bruce Taylor.

From Macquarie University Hospital, Sydney, Australia, Dr Ik Lin Tan.

From Concord Repatriation General Hospital, Sydney, Australia, Dr Todd Hardy.

From Royal Brisbane and Women's Hospital, Brisbane, Australia, Dr Pamela McCombe.

From AZ Alma Ziekenhuis, Sijsele - Damme, Belgium, Dr Danny Decoo.

From Rehabilitation and MS-Centre Overpelt and Hasselt University, Hasselt, Belgium, Dr Bart Van Wijmeersch.

From Hospital Ecoville, Curitiba, Brazil, Dr Walter Oleschko Arruda.

From Jewish General Hospital, Montreal, Canada, Dr Fraser Moore.

From CHRTR, Trois-Rivieres, Canada, Dr Stephane Charest.

From , , Dr Claudio Gobbi.

From Multiple Sclerosis Centre Kamillus-Klinik, Asbach, Germany, Dr Dieter Poehlau.

From Alexandria University Hospital, Alexandria, Egypt, Dr Karim Kotkata.

From Hospital Universitario Virgen de Valme, Seville, Spain, Dr Ricardo Fernandez BolaÒos.

From Hospital Clinico San Carlos, Madrid, Spain, Dr Celia Oreja-Guevara.

From Hospital Universitario de la Ribera, Alzira, Spain, Dr Jose Andres Dominguez.

From Hospital Ramún y Cajal, Madrid, Spain, Dr Jose C Alvarez-Cermeno.

From The Walton Centre for Neurology and Neurosurgery, Liverpool, United Kingdom, Dr Carolyn Young.

From Craigavon Area Hospital, Craigavon, United Kingdom, Dr Jamie Campbell.

From Instituto de Seguridad Social de Guatemala, Guatemala, Guatemala, Dr Alejandro Jose Diaz Jimenez.

From Veszprém Megyei Csolnoky Ferenc Kórház zrt., Veszprem, Hungary, Dr Imre Piroska.

From Jahn Ferenc Teaching Hospital, Budapest, Hungary, Dr Csilla Rozsa, Dr Krisztian Kasa.

From Semmelweis University Budapest, Budapest, Hungary, Dr Magdolna Simo.

From Peterfy Sandor Hospital, Budapest, Hungary, Dr Krisztina Kovacs.

From Josa Andr·s Hospital, Nyiregyhaza, Hungary, Dr Tunde Erdelyi.

From BAZ County Hospital, Miskolc, Hungary, Dr Attila Sas.

From Szent Imre Hospital, Budapest, Hungary, Dr Eniko Dobos.

From University of Szeged, Szeged, Hungary, Dr Cecilia Rajda.

From Assaf Harofeh Medical Center, Beer-Yaakov, Israel, Dr Shlomo Flechter.

From Bombay Hospital Institute of Medical Sciences, Mumbai, India, Dr Bhim Singhal.

From Golestan, Ahvaz, Iran, Dr Seyed Aidin Sajedi.

From University of Florence, Florence, Italy, Dr Maria Pia Amato.

From ASL3 Genovese, Genova, Italy, Dr Claudio Solaro.

From Clinical Centar-Neurology, Skopje, Macedonia, Dr Vladimir Bojkovski.

From Clinic of Neurology Clinical Center, Skopje, Macedonia, Dr Tatjana Petkovska-Boskova.

From Mater Dei Hospital, Balzan, Malta, Dr Norbert Vella.

From Hospital Angeles de las Lomas. Instituto Mexicano de Neurociencias., Huixquilucan Estado de Mexico, Mexico, Dr Eli Skromne.

From HOSPITAL KUALA LUMPUR, Kuala Lumpur, Malaysia, Dr Joyce Pauline Joseph.

From Penang General Hospital, Penang, Malaysia, Dr Jyh Yung Hor.

From Jeroen Bosch Ziekenhuis, Den Bosch, Netherlands, Dr Erik van Munster.

From Royal Hospital, Muscat, Oman, Dr Jabir Alkhaboori.

From Hospital S„o Jo„o, Porto, Portugal, Dr Maria Edite Rio.

From Central Military Emergency University Hospital, Bucharest, Romania, Dr Carmen-Adella Sirbu.

From Razi Hospital, Manouba, Tunisia, Dr Youssef Sidhom, Dr Riadh Gouider.

From Thomas Jefferson University, United States, Dr Donald McCarren.

From GF Ingrassia, Catania, Italy, Dr Clara Chisari, Dr Emanuele D'Amico, Dr Lo Fermo Salvatore.

From University G. díAnnunzio, Chieti, Italy, Dr Giovanna De Luca, Dr Valeria Di Tommaso, Dr Daniela Travaglini, Dr Erika Pietrolongo, Dr Maria di Ioia, Dr Deborah Farina, Dr Luca Mancinelli.

From CHUM MS Center and Universite de Montreal, Montreal, Canada, Dr C. Larochelle.

From Azienda Ospedaliera Universitaria, Modena, Italy, Dr Francesca Vitetta, Dr Anna Maria Simone.

From University of Melbourne, Melbourne, Australia, Dr Mark Marriott, Dr Trevor Kilpatrick, Dr John King, Dr Katherine Buzzard, Dr Ai-Lan Nguyen, Dr Chris Dwyer, Dr Mastura Monif, Dr Izanne Roos, Ms Lisa Taylor, Ms Josephine Baker.

From Azienda Sanitaria Unica Regionale Marche - AV3, Macerata, Italy, Dr Matteo Diamanti.

From Hospital Italiano, Buenos Aires, Argentina, Dr Juan Ingacio Rojas.

From University of Parma, Parma, Italy, Dr Erica Curti, Dr Elena Tsantes.

From Monash University, Melbourne, Australia, Dr Anneke van der Walt.

Administrative and technical support was provided by:

From the MSBase Administrations Ms Charlotte Sartori, Dr Sabah Quddus, Ms Eloise Hinson.

**Supplementary table 5** : Patients disposition per centre, patients with relapsing or progressive MS selected for this study.

| **Centre** |  | **Patients** |
| --- | --- | --- |
| Hospital Fernandez, Capital Federal, Argentina | | 72 |
| Clinica Modelo de Lanus, Lanus, Argentina | | 1 |
| INEBA - Institute of Neuroscience Buenos Aires, Buenos Aires, Argentina | | 80 |
| Instituto de Neurociencias Cordoba, Cordoba, Argentina | | 13 |
| Centro de Esclerosis Múltiple de Buenos Aires (CEMBA), Buenos Aires, Argentina | | 85 |
| HIGA Gral. San Martin LaPlata, La Plata, Argentina | | 11 |
| Sanatorio Allende, Cordoba, Argentina | | 11 |
| University of Western Australia, Nedlands, Australia | | 38 |
| Brain and Mind Centre, Sydney, Australia | | 191 |
| Melbourne MS Centre, Department of Neurology, Royal Melbourne Hospital, Melbourne, Australia | | 722 |
| University Newcastle, Newcastle, Australia | | 436 |
| Geelong Hospital, Geelong, Australia | | 38 |
| St Vincents Hospital, Fitzroy, Melbourne, Australia | | 20 |
| Monash Medical Centre, Melbourne, Australia | | 293 |
| Liverpool Hospital, Sydney, Australia | | 83 |
| Box Hill Hospital, Melbourne, Australia | | 673 |
| Westmead Hospital, Sydney, Australia | | 160 |
| Flinders University, Adelaide, Australia | | 202 |
| University of Queensland, Brisbane, Australia | | 220 |
| Townsville Hospital, Townsville, Australia | | 14 |
| Royal Hobart Hospital, Hobart, Australia | | 17 |
| The Alfred Hospital, Melbourne, Australia | | 165 |
| Austin Health, Melbourne, Australia | | 135 |
| Macquarie University Hospital, Sydney, Australia | | 8 |
| Concord Repatriation General Hospital, Sydney, Australia | | 24 |
| Royal Brisbane and Women's Hospital, Brisbane, Australia | | 14 |
| Cliniques Universitaires Saint-Luc, Brussels, Belgium | | 340 |
| AZ Alma Ziekenhuis, Sijsele - Damme, Belgium | | 16 |
| Universitary Hospital Ghent, Ghent, Belgium | | 145 |
| Rehabilitation and MS-Centre Overpelt and Hasselt University, Hasselt, Belgium | | 249 |
| Hospital Ecoville, Curitiba, Brazil | | 6 |
| Universidade Metropolitana de Santos, Santos, Brazil | | 101 |
| Hospital Universitario Gaffree e Guinle, Rio de Janeiro, Brazil | | 8 |
| CSSS Saint-Jérôme, Saint-Jerome, Canada | | 248 |
| Jewish General Hospital, Montreal, Canada | | 58 |
| CHUM and Universite de Montreal, Montreal, Canada | | 808 |
| CHRTR, Trois-Rivieres, Canada | | 6 |
| CISSS Chaudi?re-Appalache, Levis, Canada | | 658 |
| Neuro Rive-Sud, Quebec, Canada | | 577 |
| St. Michael's Hospital, Toronto, Canada | | 106 |
| Centro Internacional de Restauracion Neurologica, Havana, Cuba | | 70 |
| Charles University in Prague and General University Hospital, Prague, Czech Republic | | 2471 |
| Nemocnice Jihlava, Jihlava, Czech Republic | | 246 |
| Aarhus University Hospital, Arhus C, Denmark | | 96 |
| Ain Shams University, Cairo, Egypt | | 212 |
| Kasr Al Ainy MS research Unit (KAMSU), Cairo, Egypt | | 42 |
| Hospital Universitario Virgen de Valme, Seville, Spain | | 200 |
| Hospital Universitario Donostia, San Sebastián, Spain | | 127 |
| Hospital Clinico San Carlos, Madrid, Spain | | 257 |
| Hospital General Universitario de Alicante, Alicante, Spain | | 27 |
| Hospital Universitario Virgen Macarena, Sevilla, Spain | | 1170 |
| Hospital de Galdakao-Usansolo, Galdakao, Spain | | 122 |
| Hospital Germans Trias i Pujol, Badalona, Spain | | 191 |
| Hospital Universitario de la Ribera, Alzira, Spain | | 22 |
| Hospital Ramón y Cajal, Madrid, Spain | | 1 |
| Hospital Clinic de Barcelona, Barcelona, Spain | | 284 |
| University Hospital Reina Sofia, Cordoba, Spain | | 145 |
| MS Clinic, Hopital Tenon , Paris, France | | 23 |
| Craigavon Area Hospital, Craigavon, United Kingdom | | 49 |
| Royal Victoria Hospital, Belfast, United Kingdom | | 7 |
| South East Trust, Belfast, United Kingdom | | 86 |
| AHEPA University Hospital, Thessaloniki, Greece | | 14 |
| Veszprém Megyei Csolnoky Ferenc Kórház zrt., Veszprem, Hungary | | 18 |
| Jahn Ferenc Teaching Hospital, Budapest, Hungary | | 32 |
| Semmelweis University Budapest, Budapest, Hungary | | 22 |
| University of Debrecen, Debrecen, Hungary | | 86 |
| Péterfy Sandor Hospital, Budapest, Hungary | | 17 |
| Josa András Hospital, Nyiregyhaza, Hungary | | 6 |
| BAZ County Hospital, Miskolc, Hungary | | 10 |
| Szent Imre Hospital, Budapest, Hungary | | 11 |
| University of Szeged, Szeged, Hungary | | 10 |
| St Vincent's University Hospital, Dublin, Ireland | | 54 |
| Assaf Harofeh Medical Center, Beer-Yaakov, Israel | | 89 |
| Bombay Hospital Institute of Medical Sciences, Mumbai, India | | 23 |
| PGIMER, Chandigarh, India | | 9 |
| Isfahan University of Medical Sciences, Isfahan, Iran | | 699 |
| University G. d’Annunzio, Chieti, Italy | | 889 |
| Azienda Sanitaria Unica Regionale Marche - AV3, Macerata, Italy | | 330 |
| University of Florence, Florence, Italy | | 97 |
| IRCCS Mondino Foundation, Pavia, Italy | | 418 |
| Ospedali Riuniti di Salerno, Salerno, Italy | | 244 |
| University of Parma, Parma, Italy | | 284 |
| Azienda Ospedaliera di Rilievo Nazionale San Giuseppe Moscati Avellino, Avellino, Italy | | 272 |
| Azienda Ospedaliera Universitaria, Modena, Italy | | 557 |
| ASL3 Genovese, Genova, Italy | | 228 |
| Department of Medical and Surgical Sciences and Advanced Technologies, GF Ingrassia, Catania, Italy | | 1604 |
| Neurology Unit, Garibaldi Hospital, Catania, Italy | | 280 |
| Amiri Hospital, Sharq, Kuwait | | 835 |
| American University of Beirut Medical Center, Beirut, Lebanon | | 497 |
| Clinical Centar-Neurology, Skopje, Macedonia | | 2 |
| Clinic of Neurology Clinical Center, Skopje, Macedonia | | 14 |
| Mater Dei Hospital, Balzan, Malta | | 28 |
| Instituto Nacional de Ciencias Médicas y Nutrición Salvador Zubirán, Mexico City, Mexico | | 4 |
| HOSPITAL KUALA LUMPUR, Kuala Lumpur, Malaysia | | 1 |
| University Hospital Nijmegen, Nijmegen, Netherlands | | 132 |
| Francicus Ziekenhuis, Roosendaal, Netherlands | | 51 |
| Zuyderland Medical Centre, Sittard-Geleen, Netherlands | | 397 |
| Jeroen Bosch Ziekenhuis, Den Bosch, Netherlands | | 35 |
| Groene Hart Ziekenhuis, Gouda, Netherlands | | 92 |
| Medical Center Leeuwarden, Leeuwarden, Netherlands | | 8 |
| Waikato Hospital, Hamilton, New Zealand | | 31 |
| Royal Hospital, Muscat, Oman | | 26 |
| Sultan Qaboos University Hospital, Al-Khodh, Oman | | 83 |
|  | |  |
| Centro Hospitalar Universitario de Sao Joao, Porto, Portugal | | 462 |
| Central Military Emergency University Hospital, Bucharest, Romania | | 16 |
| King Fahad Specialist Hospital-Dammam, Khobar, Saudi Arabia | | 77 |
| Razi Hospital, Manouba, Tunisia | | 117 |
| KTU Medical Faculty Farabi Hospital, Trabzon, Turkey | | 575 |
| 19 Mayis University, Samsun, Turkey | | 714 |
| Hacettepe University, Ankara, Turkey | | 75 |
| Koc University, Istanbul, Turkey | | 117 |
| Dokuz Eylul University, Konak/Izmir, Turkey | | 1312 |
| Bakirkoy Education and Research Hospital for Psychiatric and Neurological Diseases, Istanbul, Turkey | | 240 |
| Haydarpasa Numune Training and Research Hospital, Istanbul, Turkey | | 302 |


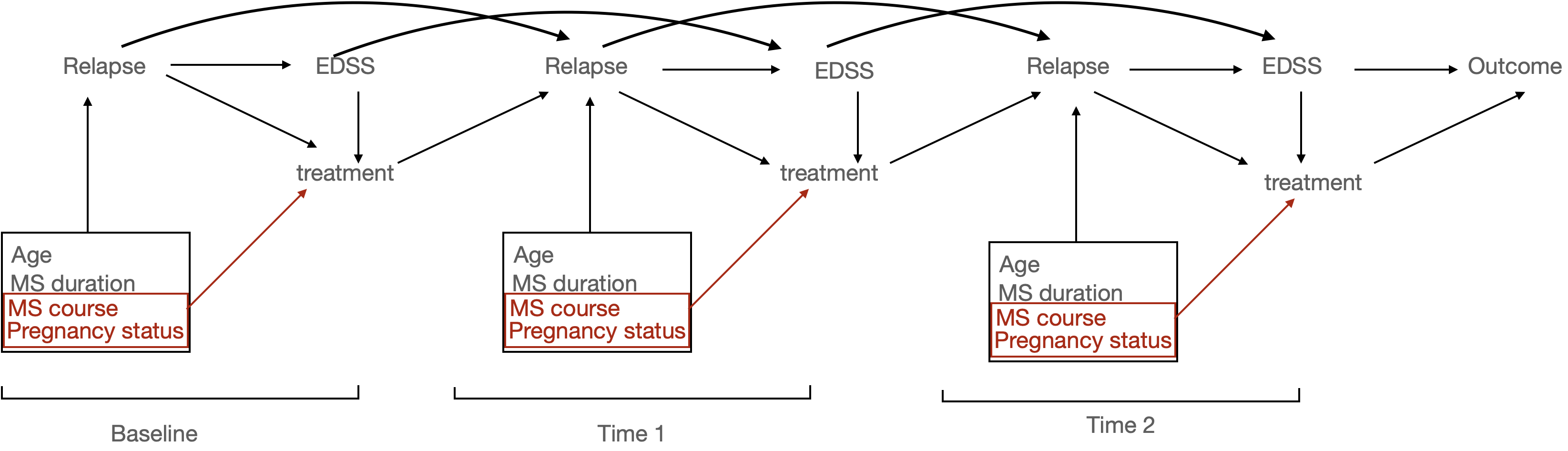


Supplementary figure 1. This figure depicts hypothetical relationships between demographic factors, EDSS, relapses and treatment for a 3 time-points follow-up. In this causal diagram, a history relapse is a confounding factor for the associations between baseline treatment and baseline EDSS while at Time 1 while a relapse at Time 1 is an intermediate between baseline treatment and EDSS at Time 1.


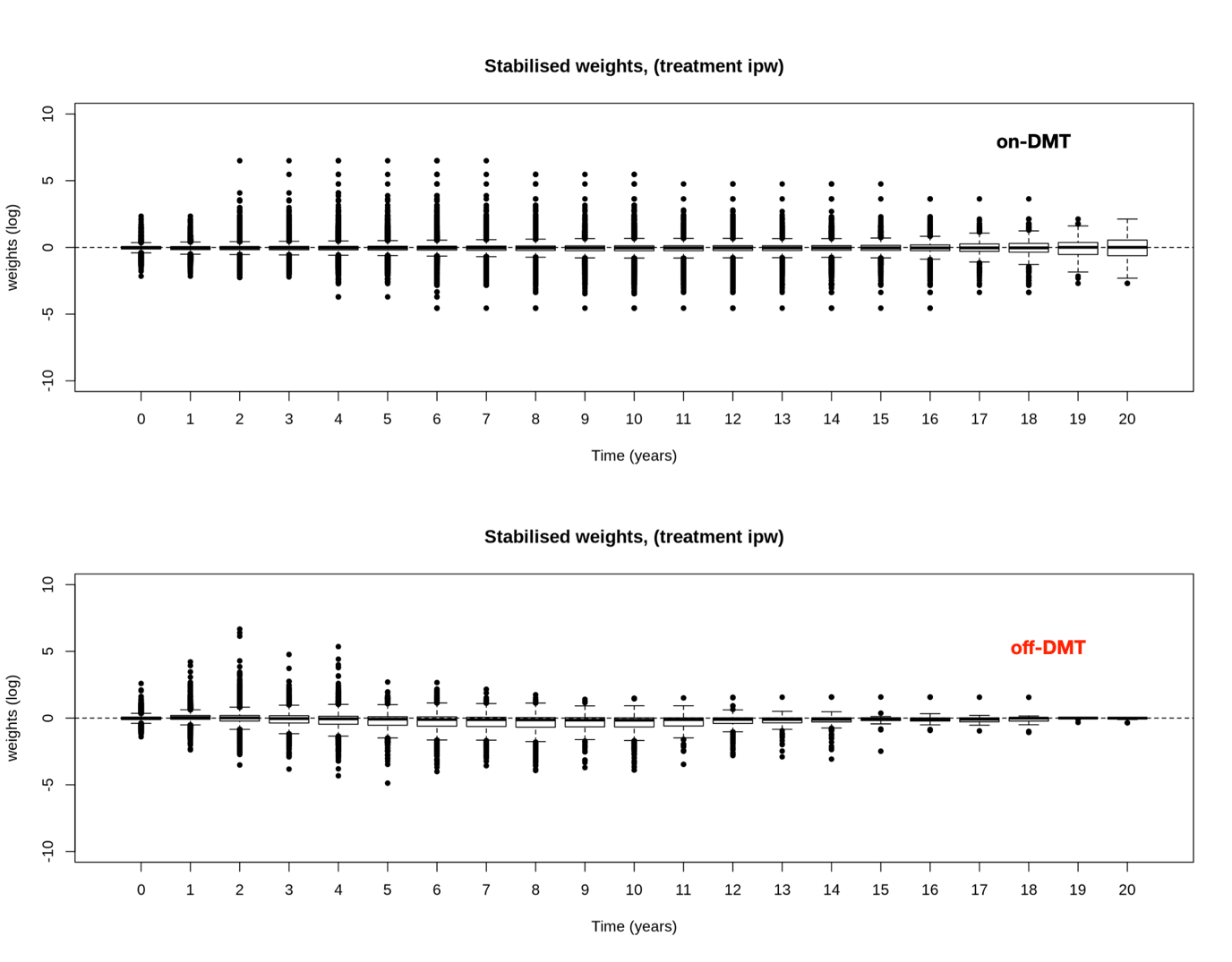


Supplementary figure 2. Weights for models of the main effects of treatment on disability improvement, disability worsening and relapses using approach A. The weights are shown at each 6-month period over up to 20 years of follow-up. The box and whisker plots show median, quartiles, and 1.5x interquartile range


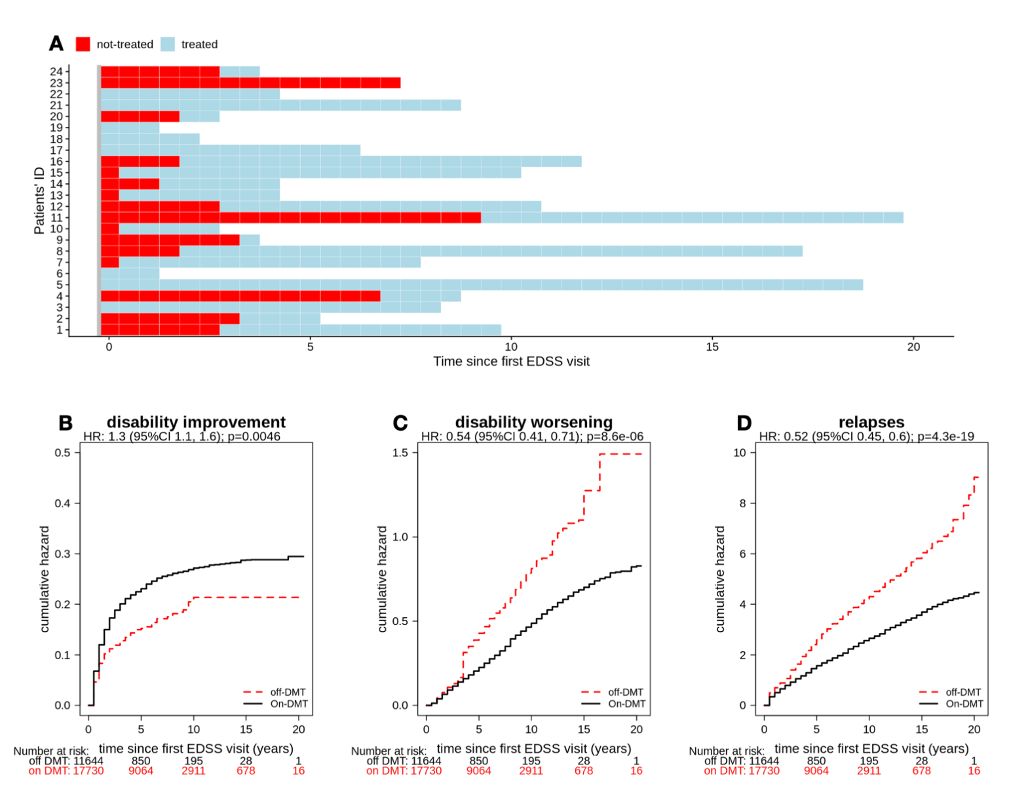


**Supplementary figure 3 (approach A), results from marginal structural model**. Participants’ first EDSS visit was used as a baseline (time 0). The time was re-set to baseline if patients shifted from “non-treated” to “treated”. Follow-up was censored at first treatment discontinuation, meaning that patients cannot contribute to the analysis a “non-treated” period after a “treated” period (**A**). The figure compares cumulative hazard of disability improvement (**B**), disability worsening (**C**) and relapses (D) between the treated and untreated pseudo cohorts.


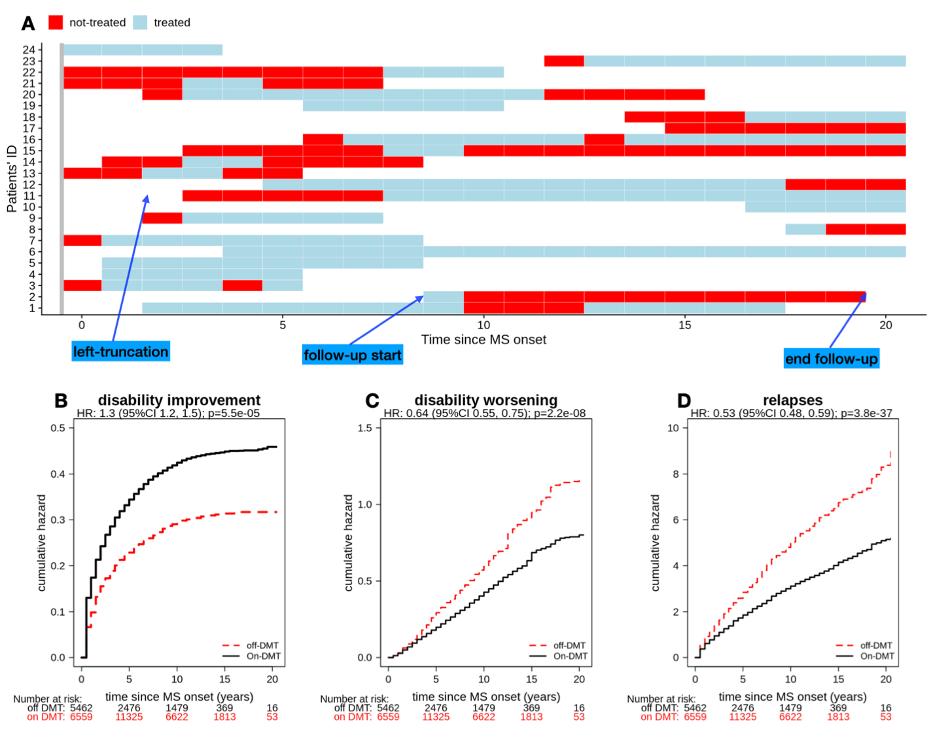


**Supplementary figure 4 (approach B), results from marginal structural model**. Patients’ MS onset date was used as a baseline without additional re-baselining of patients’ follow-up. Patients were allowed to switch freely between the treated and untreated status (**A**). The figure compares cumulative hazard of disability improvement (**B**), disability worsening (**C**) and relapses (D) between the treated and untreated pseudo cohorts.


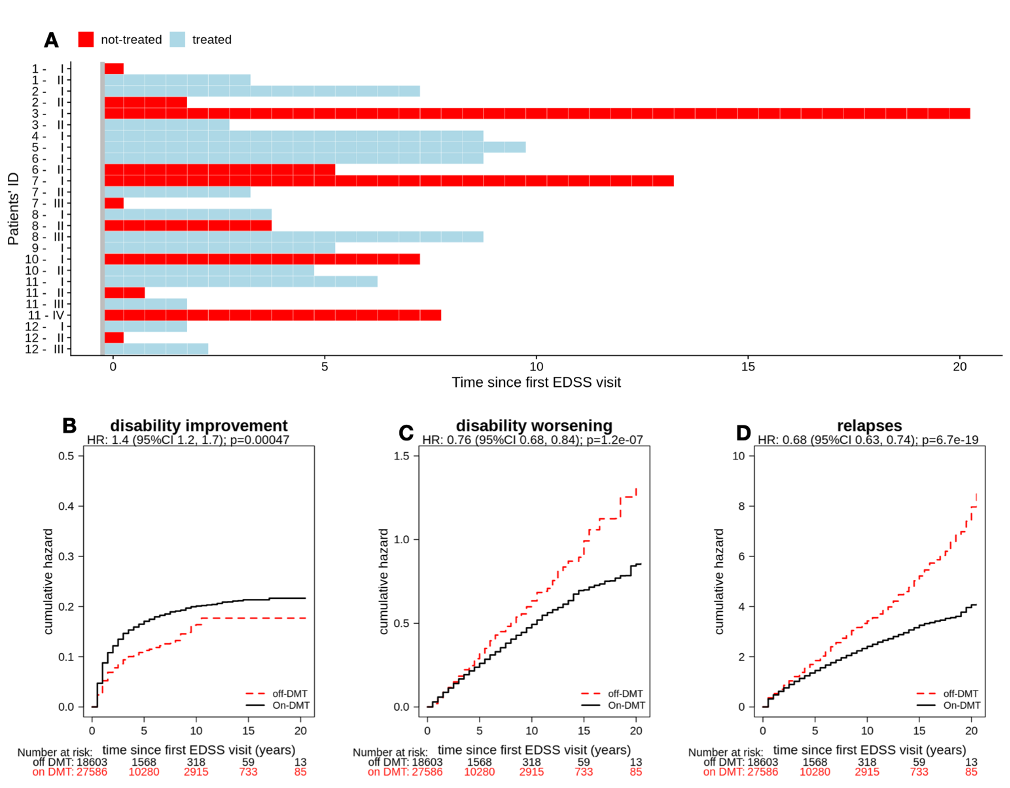


**Supplementary figure 5 (approach C), results from marginal structural model**. Patients’ first EDSS visit was used as baseline with follow-up being re-baselined at each change in treatment status. Switches in both directions between the treated and untreated status were allowed (**A**). The figure compares cumulative hazard of disability improvement (**B**), disability worsening (**C**) and relapses (D) between the treated and untreated pseudo cohorts
